# Unemployment Insurance

**Published:** 1912-10-26

**Authors:** 


### NATIONAL INSURANCE ACT DEPARTMENT.

### XXXII.—Unemployment Insurance—(*continued*).

The first condition of a sound scheme of insurance against unemployment laid down by Sir H. Llewellyn Smith, as quoted in our last week's issue, was that the scheme must be compulsory. The compulsory character of the unemployment provisions of the National Insurance Act is indicated in the first section of Part II. (Section 84) by the words "*Every* workman . . . in whose case the conditions laid down . . . are fulfilled *shall* be entitled . . . to receive unemployment benefit."

The contributory character of the scheme, the second of the essential conditions, finds expression in Section 85, which declares that "the sums required for the payment of unemployed benefit under this Act shall be derived partly from contributions by workmen . . . and partly from contributions by employers of such workmen, and partly from moneys provided by Parliament." This quotation also touches upon two of the other criteria of a sound scheme—namely, the necessity of interesting the employers in it by requiring them to take their share in the contributions, and also of the necessity of a State subvention.

The third condition is that there must be a maximum limit to the amount of benefit which can be drawn. The absolute limit as laid down in the Seventh Schedule during which unemployment benefit may be paid in the course of twelve months is fifteen weeks, and the minimum limit imposed is that benefit shall not be payable in respect of any period less than one day. Thus the unemployment fund is protected on the one hand against claims for an unusually protracted duration, and on the other hand against petty claims for small amounts which, in the aggregate, might have been a serious charge if they had not been excluded. The limitation of benefit in relation to the amount of contributions paid is equally essential, and this has found expression in the provision that no workman shall receive more unemployment benefit than in the proportion of one week's benefit for every five contributions paid. Certain concessions in the application of this rule are made to workmen who have been habitually employed in an insured trade before the commencement of the Act.

Closely analogous to the limitation of benefit payments in the matter of duration is the limitation of the actual value of the benefit. This must be such as shall not be an inducement to the unemployed workman to refrain from seeking employment. This criterion seems to have been amply secured under the Act, for the full rate of benefit is no more than seven shillings a week, and when the age of the workman is between seventeen and eighteen the benefit is at half-rate only, whilst in the case of a workman under seventeen years of age no benefit at all is payable. Furthermore no payment is to be made in respect of the first week of any period of unemployment, and the period of unemployment is not to be deemed to commence until application for benefit has been made in the prescribed manner.

The last of the conditions with which it will be possible for us to deal this week is that "the scheme should be based on the trade group, and should at the outset be partial in operation."

### Workmen in the Insured Trades.

We now proceed to indicate a little more closely the actual processes adopted in order to carry out the application of these conditions. The Act is only compulsory so far as workmen in whose case the conditions laid down in the Act are fulfilled. The first word that claims attention here is the word "workman." This is defined in Section 107 as "any person of the age of sixteen or upwards employed wholly or mainly by way of manual labour, who has entered into or works under a contract of service with an employer, whether the contract is expressed or implied, is oral or in writing." It will at once be observed that the definition excludes from the compulsory operation of the Act clerks and foremen, whose duties cannot be classed as "manual labour." There is also in the definition the same expression as in the description of employed persons for the purpose of Part I. of the Act—namely, that it refers to those who have entered into a "contract of service"—a phrase that we commented upon in an earlier issue. The first of the fundamental conditions is that the workman shall have been employed in one of the "insured trades." The "insured trades" are classed in the Sixth Schedule under seven separate headings, but they may for convenience be classed in two more comprehensive groups—namely, (1) the building group and (2) the engineering group.

There were special reasons for the selection of these trades as a commencement for unemployment insurance. They are the trades most subject to depression in times of commercial inactivity, and they are also subject to well-defined seasonal fluctuations, both of which are particularly fit subjects for insurance protection. Then, again, it so happened that almost the only data available for determining the appropriate rates of contribution for the desired benefits had reference to these two groups of trades.

It should be noted that during the passage of the Bill through Parliament certain additions were made to the list of insured trades, which in the first instance was comprised under five headings only. The two additional trades were (1) ironfounding and (2) sawmilling. Several modifications were made in the description of the trades specified; for instance, the building trade was originally described as "the construction, alteration, repair, decoration, or demolition of any building or any part thereof." This description is retained fundamentally in the Act, but with the added qualification that it is to include the manufacture of any fittings of wood of a kind commonly made in builders' workshops or yards, so that a workman is required to be insured if he is engaged in work connected with building operations in precisely the same manner as he would be if he were actually employed on the building in course of erection or alteration or repair. Within the main group of building trades, to which we referred, is included the construction of works such as railways, docks, harbours, canals, bridges, and so forth, as also the reconstruction and alteration of works of the same general description.

The engineering section is represented by the ship-building and mechanical engineering trades, the latter including the manufacture of ordnance and firearms. Within the same group ironfounding and the construction of vehicles must be mentioned.

Insurance Depends on Nature of Work.

In connection with this description of the insured trades a very important provision of the Act has to be borne in mind—namely, that in determining whether any trade in which a workman is engaged is or is not an insured trade the point to be considered is not whether the business of the employer is one of the insured trades, but regard is to be given to the nature of the work on which the workman is actually engaged.

Thus it would appear that a workman engaged as an engineer to mind and repair the engine at a mill or factory, which clearly is not included in the operation of the unemployment section, would by reason of the nature of the work on which he is actually engaged have to be insured against unemployment, as well as under the health insurance provisions.

This is doubtless a very wise general precaution, but there are many cases where it is known to operate harshly, for in cases similar to the one we have cited it frequently happens that the workman's appointment may be considered to all intents and purposes as a permanency with practically no risk of unemployment for which benefit could be drawn, and thus the contributions of employer and worker are devoted to an object which, in the circumstances, is unnecessary.

**NOTE.—Questions and Correspondence on all doubtful points are invited, and should be addressed to the Editor, The Hospital, 28 Southampton Street, Strand, W.C., and marked "Insurance."**

